# Identification of two novel breast cancer loci through large-scale genome-wide association study in the Japanese population

**DOI:** 10.1038/s41598-019-53654-9

**Published:** 2019-11-22

**Authors:** Siew-Kee Low, Yoon Ming Chin, Hidemi Ito, Keitaro Matsuo, Chizu Tanikawa, Koichi Matsuda, Hiroko Saito, Mika Sakurai-Yageta, Naoki Nakaya, Atsushi Shimizu, Satoshi S. Nishizuka, Taiki Yamaji, Norie Sawada, Motoki Iwasaki, Shoichiro Tsugane, Toshiro Takezaki, Sadao Suzuki, Mariko Naito, Kenji Wakai, Yoichiro Kamatani, Yukihide Momozawa, Yoshinori Murakami, Johji Inazawa, Yusuke Nakamura, Michiaki Kubo, Toyomasa Katagiri, Yoshio Miki

**Affiliations:** 1 Cancer Precision Medicine Center, Japanese Foundation for Cancer Research, Tokyo, Japan; 2 Laboratory for Statistical Analysis, RIKEN Center for Integrative Medical Sciences, Yokohama, Japan; 3 Laboratory for Genotyping Development, RIKEN Center for Integrative Medical Sciences, Yokohama, Japan; 4 Division of Cancer Information and Control, Aichi Cancer Center Research Institute, Nagoya, Japan; 5 Division of Cancer Epidemiology and Prevention, Aichi Cancer Center Research Institute, Nagoya, Japan; 6 Department of Epidemiology, Nagoya University Graduate School of Medicine, Nagoya, Japan; 7 Laboratory of Genome Technology, Human Genome Center, The University of Tokyo, Tokyo, Japan; 8 Division of Molecular Pathology, The Institute of Medical Science, The University of Tokyo, Tokyo, Japan; 9 Graduate school of Frontier Sciences, The University of Tokyo, Tokyo, Japan; 10 Department of Genetic Diagnosis, The Cancer Institute of JFCR, Tokyo, Japan; 11 Tohoku Medical Megabank Organization, Tohoku University, Sendai, Japan; 12 Iwate Tohoku Medical Megabank Organization, Iwate Medical University, Iwate, Japan; 13 Division of Epidemiology, National Cancer Center, Tokyo, Japan; 14 Center for Public Health Sciences, National Cancer Center, Tokyo, Japan; 15 Department of International Island and Community Medicine, Kagoshima University Graduate School of Medical and Dental Sciences, Kagoshima, Japan; 16 Department of Public Health, Nagoya City University Graduate School of Medical Sciences, Nagoya, Japan; 17 Department of Preventive Medicine, Nagoya University Graduate School of Medicine, Nagoya, Japan; 18 Department of Oral Epidemiology, Graduate School of Biomedical and Health Sciences, Hiroshima University, Hiroshima, Japan; 19 Department of Molecular Cytogenetics, Tokyo Medical & Dental University, Tokyo, Japan; 20 Department of Molecular Genetics, Medical Research Institute, Tokyo Medical & Dental University, Tokyo, Japan; 21 Bioresource Research Center, Tokyo Medical & Dental University, Tokyo, Japan; 22 Division of Genome Medicine, Institute for Genome Research, Tokushima University, Tokushima, Japan

**Keywords:** Breast cancer, Cancer genomics

## Abstract

Genome-wide association studies (GWAS) have successfully identified about 70 genomic loci associated with breast cancer. Owing to the complexity of linkage disequilibrium and environmental exposures in different populations, it is essential to perform regional GWAS for better risk prediction. This study aimed to investigate the genetic architecture and to assess common genetic risk model of breast cancer with 6,669 breast cancer patients and 21,930 female controls in the Japanese population. This GWAS identified 11 genomic loci that surpass genome-wide significance threshold of *P* < 5.0 × 10^−8^ with nine previously reported loci and two novel loci that include rs9862599 on 3q13.11 (*ALCAM*) and rs75286142 on 21q22.12 (*CLIC6-RUNX1*). Validation study was carried out with 981 breast cancer cases and 1,394 controls from the Aichi Cancer Center. Pathway analyses of GWAS signals identified association of dopamine receptor medicated signaling and protein amino acid deacetylation with breast cancer. Weighted genetic risk score showed that individuals who were categorized in the highest risk group are approximately 3.7 times more likely to develop breast cancer compared to individuals in the lowest risk group. This well-powered GWAS is a representative study to identify SNPs that are associated with breast cancer in the Japanese population.

## Introduction

Breast cancer is the most common malignancy among women worldwide. Based on the report of Cancer Statistics in Japan 2018^1^, it is estimated that the incidence of breast cancer will rise to 86,500 in the year 2018, which comprise approximately 20% of all female cancers. Breast cancer is also the fifth leading cause of cancer death among women in Japan, with an estimated death of 14,285 in the year of 2017. Despite better 5-year survival rates for breast cancer compared to other malignancies, the age-adjusted incidence and mortality rate in Japan has increased steadily since the 1970s. Hence, predictive genetic markers and early detection screening methods to identify individuals at risk of breast cancer are crucial to reduce breast-cancer associated death.

Breast cancer is a complex polygenic disease with diverse risk factors that include lifestyle and genetic mutations. Common mutations linked to breast cancer include highly-penetrant *BRCA1* and *BRCA2* genes, moderate effect size genes (*CHEK2*, *PALB2*, *PTEN* and *ATM*) as well as common variants conferring small effect sizes^2–7^. A total of 28 genome-wide association studies (GWAS) showing association with breast cancer risk have been published^8^. These studies successfully identified common variants in 70 genetic loci from diverse worldwide populations: Europe (70 loci), East Asians (8 loci), Africans (3 loci), Latinos (2 loci) and Ashkenazi Jews (1 loci)^9–35^. Risk loci and risk variants differ across different populations due to several possible reasons. These include insufficient statistical power in individual studies, complexity in linkage disequilibrium, and differences in allele frequencies as well as environmental exposure. Notably, studies have indicated the importance of carrying out regional GWAS to identify specific genetic risk factors that are associated with complex disease, which could facilitate better risk assessment in the regional clinical settings^36^. Our group has published two breast cancer GWAS: The first reported association of chromosome 10q26 (*FGFR2*) and 16q12 (*TOX-LOC643714*) to breast cancer while the second showed association of 3q25.1 (*SIAH2*) with hormonal-positive breast cancer in the Japanese population^37,38^. The size of our current study is three times that of our previous two studies^37,38^. To the best of our knowledge, it is the largest and most well-powered GWAS to date that aims to investigate the genetic architecture as well as to assess the common genetic risk model of breast cancer in the Japanese population.

## Results

### Evaluation of two GWAS with samples obtained from Phase I-II and Phase III Biobank Japan

Two sets of GWAS were performed in this study with samples obtained from Phase I-II and Phase III Biobank Japan that consist of 6,669 breast cases and 21,930 female controls.

For sample quality control, identity-by-state analysis was carried out to assess close relatedness in the sample population, no samples were removed as all the samples were independent from each other (Data not shown). Subsequently, principal component analysis (PCA) was performed to assess population substructure of the sample populations. PCA revealed that case and control subjects that participate in this study were clustered into two major clusters, the mainland (Hondo) cluster and the Ryuukyu (southern island of Japan) cluster^39^ (Supplementary Fig. 1). Association analyses were carried out by incorporating principal components as covariates to avoid the bias effects from population substructure. The quantile-quantile (Q-Q) plot and the genomic inflation factor (λ_GC_) of the test statistic for the GWAS of Phase I-II and Phase III were 1.202 and 1.019, respectively (Supplementary Fig. 2). As λ_GC_ value increase correspondingly with sample size, the λ_GC_ value adjusting to a sample size of 1000 was evaluated for GWAS of Phase I-II^40^. The adjusted λ_1000_ value was 1.025, indicating a low possibility of false-positive association by population stratification. The two GWAS studies were subsequently combined by meta-analysis after whole-genome imputation and quality control. A total of 4,946,503 SNPs was evaluated to identify common genetic variations that are associated with breast cancer susceptibility. The Manhattan plot of the whole-genome meta-analysis was plotted by using –log_10_(*P*-value) against chromosome location (Supplementary Fig. 3).

### Identification of two novel loci associated with- breast cancer patients

**Figure 1:**
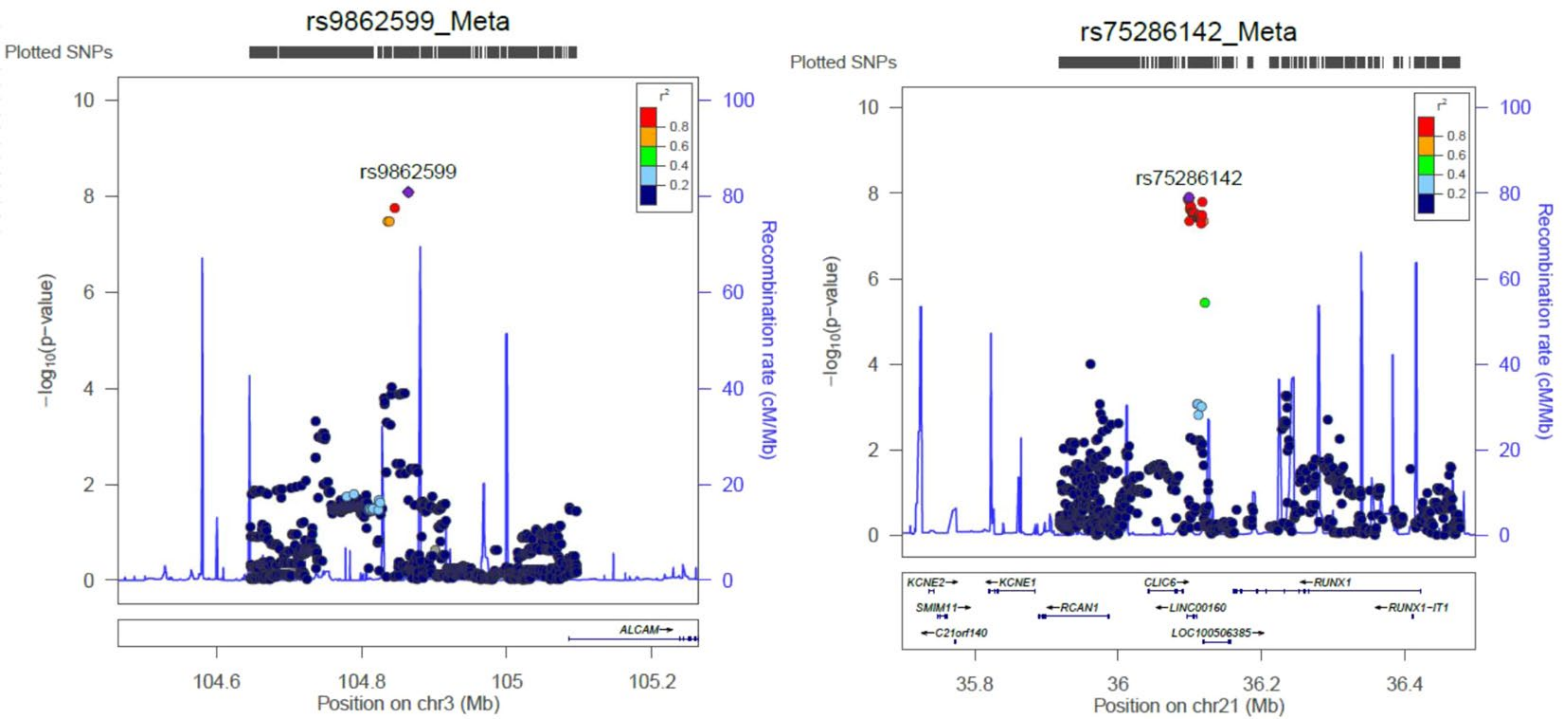
Regional plot for two novel associated loci that include 3q13.11 (ALCAM) and 21q22.12 (CLIC6-RUNX1).

This study identified a total of 11 genomic loci that surpassed the genome-wide significance level with *P*-value threshold of 5.0 × 10^−8^ to be associated with breast cancer. These loci included 2q33.1 (*TRAK2-ALS2CR11*), 3q13.11 (*ALCAM*), 3q25.1 (*SIAH2*), 5p12 (*FGF10-MRPS30*), 5q11.2 (*MAP3K1*), 6q25.1 (*CCDC170-ESR1*), 10q26.13 (*FGFR2*), 12p11.22 (*PTHLH*), 12q24.21 (*UBA52P7*), 16q12.1 (2 independent SNPs on *TOX3-CASC16*) and 21q22.12 (*CLIC6-RUNX1*) shown in Table 1. We compared them with previously reported breast cancer susceptibility loci that are identified from GWAS of multiple ethnicities, European, East Asian, African and Ashkenazi Jews, and found that nine loci identified from this study were previously reported while 3q13.11 (*ALCAM*) and 21q22.12 (*CLIC6-RUNX1*) are novel associated loci that surpassed genome-wide significance threshold (Table 1 and Fig. 1). In addition, a total of 40 previously reported SNPs from 37 genomic loci remained to be suggestively associated with the range of *P-*value from 4.88 × 10^−2^ to 6.01 × 10^−6^ in this GWAS study (Supplementary Table 2).

**Table 1:** SNPs that surpassed genome-wide significance threshold after meta-analysis of GWAS I + II.

CHR	MARKER	POS	Risk Allele	Reference Allele	RAF_ Case	RAF_ Control	pMeta	OR	95% CI (lower)	95% CI (upper)	Gene/Nearby gene
2	rs2540431	202367621	T	G	0.330	0.313	2.38E-08	1.131	1.083	1.181	*TRAK2-ALS2CR11*
*3	rs9862599	104864331	G	T	0.092	0.078	8.11E-09	1.234	1.149	1.325	*ALCAM*
3	rs1838337	150479816	G	T	0.657	0.627	1.33E-08	1.128	1.082	1.176	*SIAH2*
5	rs7701466	44663137	T	C	0.518	0.492	2.22E-10	1.138	1.094	1.184	*FGF10-MRPS30*
5	rs79160707	56052938	T	C	0.113	0.100	4.98E-08	1.200	1.124	1.282	*MAP3K1*
6	rs6900157	151954127	C	T	0.305	0.269	5.01E-10	1.147	1.099	1.198	*CCDC170-ESR1*
10	rs2912778	123338654	G	A	0.560	0.507	1.43E-19	1.201	1.155	1.250	*FGFR2*
12	rs805583	28152993	G	A	0.790	0.760	2.86E-10	1.170	1.114	1.228	*PTHLH*
12	rs10744856	115835385	G	A	0.664	0.626	1.04E-08	1.130	1.083	1.178	*UBA52P7*
16	rs3803662	52586341	A	G	0.575	0.523	1.36E-16	1.183	1.136	1.231	*TOX3-CASC16*
16	rs4784227	52599188	T	C	0.279	0.235	2.18E-24	1.271	1.214	1.331	*TOX3-CASC16*
*21	rs75286142	36098645	G	C	0.198	0.178	1.28E-08	1.158	1.101	1.218	*CLIC6-RUNX1*

RAF: risk allele frequency; P-meta: Meta-analysis of GWAS I + II; OR referred to reference allele: odds ratio; CI: confidence interval; *Novel loci from this GWAS.

The association of the 12 genome-wide significant SNPs comprises breast cancer patients of various cancer subtypes. To evaluate the effect of cancer subtype towards SNP association, a subset analysis was performed for ER+, PR+ and HER2+ respectively (Supplementary Table 3). In general, breast cancer subtype associations for all SNPs were weaker compared to the cumulative cohort (Supplementary Table 3). Validity of the associations were confirmed through permutation, with the permutated *P*-values of combined cohorts for all SNPs in different subtypes showing less than 5% false positives (Supplementary Table 3). However, the effect size shows a similar trend across different cancer subtypes. The results suggest that association of the 12 genome-wide significant SNPs show a consistent trend across different cancer subtypes and the lack of genome-wide significance is due to insufficient statistical power.

In our replication study, none of the 12 genome-wide significant SNPs was statistically significant after adjusting for multiple testing at *P* < 0.002 (0.05/22 independent tests). Despite this, the 12 SNPs showed similar effect size trends with Phase I-II and III cohorts. In addition, the inclusion of the replication cohort improved association *P-*values in the meta-analysis combining GWAS and replication studies (Supplementary Table 4). For example, the association of rs9862599 and rs75286142 on *ALCAM* and *CLIC6-RUNX1* were not replicated after considering multiple testing. However, the combined *P*-values of rs9862599 (*P* = 1.14 × 10^−8^; OR = 1.216; 95% CI = 1.136–1.301) and rs75286142 (*P* = 2.42 × 10^−9^; OR = 1.157; 95% CI = 1.103–1.214) imply an established link between these SNPs and breast cancer susceptibility (Table 2 and Fig. 1).

**Table 2:** Validation study of the two novel loci.

CHR	MARKER	POS	Gene	Stage	Risk_ Allele	Reference_ Allele	RAF_ Case	RAF_ Ctrl	*P*-value	OR	L95	U95	P_Hetero
3	rs9862599	104864331	*ALCAM*	GWAS1	G	T	0.089	0.076	2.95E-06	1.215	1.119	1.319	
3				GWAS2	G	T	0.101	0.081	3.86E-04	1.295	1.123	1.495	
3				Meta-1	G	T	—	—	8.11E-09	1.234	1.325	1.149	
3				Replication	G	T	0.086	0.081	5.58E-01	1.065	0.864	1.313	
3				Meta-2	G	T	—	—	1.44E-08	1.216	1.136	1.301	0.318
21	rs75286142	36098645	*CLIC-RUNX1*	GWAS1	G	C	0.200	0.176	2.36E-07	1.164	1.100	1.232	
21				GWAS2	G	C	0.199	0.18	2.49E-02	1.135	1.017	1.267	
21				Meta-1	G	C	—	—	1.28E-08	1.158	1.218	1.101	
21				Replication	G	C	0.192	0.172	7.36E-02	1.148	0.987	1.336	
21				Meta-2	G	C	—	—	2.42E-09	1.157	1.103	1.214	0.918

*P*-value for meta-analysis: Inverse-variance meta-analysis method; *P*-value for heterogeneity: Cochran’s Q-test; OR is referred to reference allele: odds ratio; L95: lower 95% confidence interval; U95: upper 95% confidence interval.

### Pathway analysis identified two significant pathways associated with breast cancer

Pathway analysis that incorporated whole-genome imputed SNPs to MAGENTA software have identified two significant pathways to be associated with breast cancer. The first associated pathway is dopamine receptor medicated signaling pathway (GSEA *P*-value = 8.10 × 10^−5^, FDR = 2.90 × 10^−3^) from Panther database that encompasses with genes mostly from the *CLIC*, *EPB41* and *FRMD* families (Supplementary Table 5). The second pathway is protein amino acid deacetylation from GOTERM database (GSEA *P*-value = 1.00 × 10^−4^, FDR = 3.72 × 10^−2^), which mostly consist of genes from the *SIRT* family (Supplementary Table 5).

### Association of rs2540431-linked SNPs with lower expression of *CASP8* and *ALS2CR12* in breast mammary tissue

In order to assess the effects of SNPs with the expression of the nearby genes within the locus, eQTL analyses were performed for the 12 SNPs that surpassed the genome-wide significant threshold by using GTEx Portal, focusing with breast mammary tissue. The analysis also included all LD-linked (LD >  = 0.8) SNPs as well. eQTL was detected for rs2540431-linked SNPs rs2714486 and rs2540334 (Supplementary Fig. 4A,B). No eQTL was detected for rs2540431 itself. The risk allele rs2714486-A is correlated with lower expression of *CASP8* (*P* = 4.2 × 10^−12^; Normalized effect size = −0.44) and higher expression of *ALS2CR12*(*P* = 5.2 × 10^−7^; Normalized effect size = 0.35) in breast mammary tissue (Supplementary Fig. 4A). The same trend was observed for risk allele rs2540334-T with lower expression of *CASP8* (*P* = 6.9 × 10^−12^; Normalized effect size = −0.43) and higher expression of *ALS2CR12*(*P* = 7.8 × 10^−7^; Normalized effect size = 0.34) (Supplementary Fig. 4B)

### Calculation of weighted genetic risk score (wGRS) with SNPs significantly associated with breast cancer risk

wGRS was calculated to evaluate the cumulative effect of the significantly associated SNPs with breast cancer risk. The 12 SNPs that surpassed *P* < 5.0 × 10^−8^ were selected and their corresponding weight were calculated to be incorporated into the regression model; these SNPs include rs2540431 (0.08853), rs9862599 (0.18249), rs1838337 (0.11120), rs7701466 (0.10993), rs79160707 (0.18772), rs6900157 (0.17682), rs2912778 (0.19709), rs805583 (0.16442), rs10744856 (0.12644), rs3803662 (0.12069), rs4784227 (0.18744) and rs75286142 (0.15262). The risk score groups were divided into 5 categories, odds ratio of each category increased concordantly to the level of risk score. Individuals (4% of cases and 2% of controls) in group 5, who carry the most risk alleles, have approximately 3.7 times higher risk to develop when comparing group 1 as reference with AUC of 0.593 (Supplementary Table 6 and Supplementary Fig. 5). After genotyping the selected 12 SNPs in the replication set from Aichi Cancer Centre, the same wGRS model was used, and individuals categorized in group 5 was 4.7 times higher risk comparing with group 1 with the AUC of 0.595 (Supplementary Table 6 and Supplementary Fig. 5).

## Discussion

This large-scale GWAS, which utilizes a total of 7,650 breast cancer cases and 23,324 female controls, validated nine previously reported loci and suggested two novel loci to be associated with breast cancer in the Japanese population.

Among the previously reported loci, *FGFR2* on chr10q26.13 and *TOX3-CASC16* on chr16q12.1 are the two most significant associated breast cancer susceptible loci across different populations, followed by *MAP3K1* on chr5q11.2 and *CCDC170-ESR1* on chr6q25.1. The locus of *FGFR2* carries a significant disease burden, contributing approximately 16% of all breast cancers^41^. *FGFR2* encodes for fibroblast growth factor receptor type 2, a receptor tyrosine kinase that play a role in growth and differentiation of cells in various tissues. Recent systems biology approach identified SPDEF, ERα, FOXA1, GATA3 and PTTG1 as master regulators of FGFR2 signaling and demonstrated that ERα occupancy responds to FGFR2 signaling^42^. Another follow-up study indicated that risk alleles of SNPs on *FGFR2* augment silencer activity after map to transcriptional silencer elements and the presence of risk variants results in reduced FGFR2 expression and increased estrogen responsiveness^43^.

Another susceptibility locus identified in our study is located on chr16q12.1, close to *TOX3* and *CASC16* genes. TOX3 was reported to bind with BRCA1 promoter and negatively regulates BRCA1 expression^44^. Ectopic expression of *TOX3* is associated with tumor progression in breast cancer mouse model^44^. In addition, hypomethylation of the promoter upregulate *TOX3* luminal subtype breast cancer^45^. Taken together, both genetic and epigenetic factors play a role in TOX3 overexpression in breast cancer.

Mitogen-Activated Protein Kinase Kinase Kinase 1 (MAP3K1), is a serine/threonine kinase that involved in the mitogen-activated protein kinase (MAPK) pathway that involves Ras, Raf, Mek, and Erk. MAPK cascade is known to be an important pathway for cancer cell survival, dissemination, and resistance to drug therapy^46^. Lastly, *ESR1* encode for ER-alpha known to acts as a transcriptional regulator by interacting with estrogen and other coactivator proteins. Interestingly, previous study has reported that neoplastic *ESR1*–*CCDC170* fusions is related to a more aggressive subset of ER + breast cancer^47^.

The first novel SNP, rs9862599, identified to be suggestively associated with breast cancer from this GWAS is located on chromosome 3q13.11 near to the 5′ end of *ALCAM* gene. *ALCAM* encode for Activated Leukocyte Cell Adhesion Molecule, which is a glycoprotein that binds to T-cell differentiation antigen CD6 as well as plays a role in the process of cell adhesion and migration^48^. Decreased expression of ALCAM protein is reported as an indicator of poor prognosis in breast cancer^49^. The second novel SNP, rs75286142, is located on *CLIC6-RUNX1*. CLIC6 is one of the family members of chloride intracellular channels and CLIC6 expression profile was shown to be altered in breast cancer^50^. Runx1, a transcription factor, regulates various physiological processes that include cell proliferation, survival, differentiation and cell cycle progression. Importantly, RUNX1 somatic mutations were found in ER+, luminal subtype of breast cancer and indicate a tumor suppressor role for RUNX1^51,52^. Further *in-silico* or functional analysis should be carried out to further investigate the effect of the identified SNP to *ALCAM* and *RUNX1* gene as well as the crosstalk in between germline variations and somatic mutations in these breast cancer-associated genes.

Pathway analysis identified two pathways, dopamine receptor mediated signaling pathway and protein amino acid deacetylation, to be associated with breast cancer in this GWAS study. Dopamine receptor mediated signaling pathway consists of Chloride intracellular ionic channels family (CLIC1-6), Proteins of the 4.1 family (EPB41), Serine/threonine-protein phosphatase family (PPP1C) and FERM Domain Containing (FRDM) family. Among these gene sets, there are substantial reports about the involvement of the CLIC protein in tumorigenic process^53^. For instance, the expression of CLIC4 transcript is regulated by p53 and tumor necrosis factor α as well as related to Myc-induced apoptosis^54,55^. Additionally, CLIC1 protein levels were detected to increase in multiple cancers^53^. The second associated pathway, protein amino acid deacetylation, consist of SIRT and HDAC families. Sirtuins (SIRT1-7) play a significant role in cancer by regulating cancer-associated metabolism, modifying tumor microenvironment and affecting the response to genomic instability^56^. In breast cancer, besides SIRT6 that shows to have increased expression and act as oncogene, SIRT1, SIRT2, SIRT3 and SIRT4 exhibits to have reduced expression and act as tumor suppressor genes^56^. Although SNPs in these gene sets showed only moderate association with breast cancer, gene-set enrichment *P-*value indicated their cumulative association might be of significant for further investigations.

As an apical caspase, CASP8 functions to initiate a caspase cascade upon receipt of apoptosis signaling from a death receptor–ligand interaction^57^. In addition to its central role in apoptosis, CASP8 also plays a number of non-apoptotic roles in cells, namely promoting activation NFκB signaling, regulating autophagy and altering endosomal trafficking, and enhancing cellular adhesion and migration^57^. The role of CASP8 varies and is highly dependent on cellular context^57^. Based on the *in silico* GTEx eQTL analysis, the risk allele of rs2714486-A and rs2540334-T is correlated with lower expression of CASP8. GWAS data show that both risk alleles are more frequent in breast cancer patients compared to healthy controls. All factors considered, our data suggests that CASP8 plays an apoptotic role in breast cancer, suppressing tumor malignancy. The *ALS2CR12* gene product is a structural component of the sperm flagellum^58^. With no previous reports linking it to breast cancer, this makes *ALSCR12* a weak candidate for breast cancer susceptibility.

The AUC-value from wGRS analysis by utilizing 12 SNPs is 0.593, which indicates the current prediction model required further improvement by identifying additional markers that are associated with breast cancer susceptibility. Nevertheless, this well-powered GWAS is a representative study for the Japanese population to identify common genetic variations that are associated with breast cancer.

## Methods

### Participants in this study

Breast cancer case samples for the discovery study were recruited from the Biobank Japan (Phase I to III, http://biobankjp.org). Biobank Japan collaboratively collects and stores DNA and serum samples throughout Japan. For the discovery set, a total of 5,272 and 1,397 breast cancer patients were recruited from Phase I-II and Phase III Biobank Japan Project, respectively^59^. In the Phase I-II study, there were 2412 estrogen receptor (ER+), 2010 progesterone receptor (PR+) and 2059 human epidermal growth factor receptor (HER+) breast cancer patients. In the Phase III study, there were 998 ER+, 790 PR+ and 1143 HER+ breast cancer patients. As for control, genotyping information of 16,496 and 5,434 female individuals who do not have cancer history from population-based cohorts of the Tohoku Medical Megabank organization (ToMMo) (http://www.megabank.tohoku.ac.jp/english/), Iwate Tohoku Medical Megabank Organization (IMM), Japan Multi-Institutional Collaborative Cohort (J-MICC) Study and the Japan Public Health Center-based Prospective (JPHC) Study^60^, and a hospital-based cohort of Biobank Japan were collected, respectively.

To validate the associations identified from GWAS, a total of 981 breast cancer cases and 1,394 females were collected from Aichi Cancer Centre. The detailed sample demographic and clinical parameters are summarized in Supplementary Table 1.

All participating studies obtained informed consent from all participants by following the protocols approved by their institutional ethical committees before enrollment. The ethical committees from the Institute of Medical Science, the University of Tokyo, RIKEN Center for Integrative Medical Sciences, Tohoku Medical Megabank organization, Iwate Medical University, National Cancer Center, and Aichi Cancer Center have approved this project.

### GWAS, quality control and genotype imputation

For genome-wide genotyping in the discovery study, all Phase I-II and Phase III subjects were genotyped by Illumina HumanOmniExpress v1.1. To perform sample quality control, samples with call rates < 98% were excluded from the study. We evaluated cryptic relatedness of our samples using identity-by-state. To assess population stratification, principal component analysis (PCA) by using EIGENSTRAT software (ver3.0) was carried out to compare the distribution of principal component scores between samples with four major reference population obtained from the HapMap Database that consist of Europeans (represented by Utah Residents (CEPH) with Northern and Western European Ancestry, CEU), Africans (represented by Yoruba in Ibadan, YRI) and East Asian (represented by Japanese from Tokyo, JPT, and Han Chinese in Beijing, CHB)^61^. The top two principal components that could distinguish the clusters were used to produce scatter plot for the evaluation of distribution (Supplementary Fig. 1). Based on the PCA, 3 outliers were excluded from Phase III GWAS. For SNP quality control, SNPs with call rate < 0.99, SNPs that deviated from Hardy-Weinberg equilibrium among control samples at the threshold of 1.0 × 10^−6^, non-polymorphic SNPs, and SNPs from chromosome X, Y as well as mitochondrial SNPs were excluded from further study.

**Figure 2:**
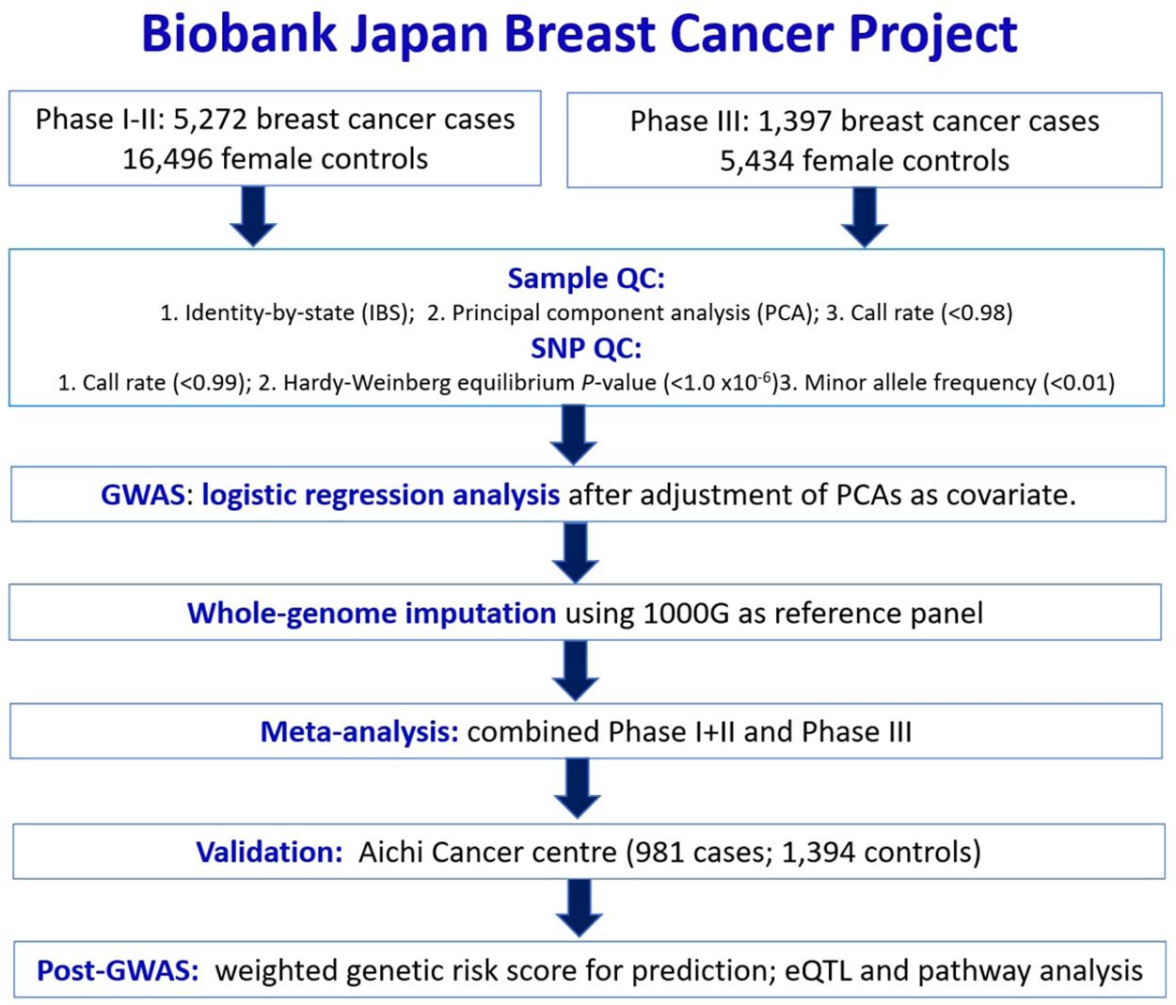
Schematic study workflow for the Biobank Japan Breast Cancer project.

Statistical analysis for both case-control GWAS, Phase I-II and Phase III, were performed by using logistic regression analysis by incorporating associated principal components as covariates. Quantile-quantile plot (Q-Q plot) of each GWAS was constructed between observed *P*-value versus expected *P-*value to evaluate potential population substructure (Supplementary Fig. 2). The genomic inflation factor (λ_GC_) values were calculated to evaluate the deviation of the GWAS distribution from the null distribution. Since inflation factor scales with sample size and considering our large sample size, λ_1000_ was also calculated. Calculation was done as previously described^62^. We used Haploview 4.2 to visualize all SNP association *P*-values in a Manhattan plot, expressed as –log_10_(*P*-value) against chromosome position (Supplementary Fig. 3)^63^. Whole genome imputation analysis was performed to infer missing genotypes that are not included in the genotyping SNP array. The 1000 G Phase I integrated release version 3 from East Asian descendant that include Japanese from Tokyo, Chinese from Beijing and Chinese from Southern China was used as a reference for this imputation analysis. In brief, SNPs with allele frequency differences that > 0.16 between GWAS and reference panel were excluded. Haplotypes of the samples were phased by using MaCH1.0 before imputation analysis was carried out referring to 1000 G reference panel with map crossover and error rates using 20 iterations of the Markov chain by using Minimac (2013.7.17) software^64,65^. Association study of the imputed genotype dosage was carried out by using mach2dat (ver1.2.4)^66^. Stringent imputation quality (R^2^) threshold was applied by excluding SNPs with R^2^ < 0.9 for further studies. To combine Phase I-II GWAS and Phase III GWAS, whole-genome meta-analysis was carried out by using Inverse-variance meta-analysis method. The schematic study workflow is summarized in Fig. 2. To address the effect of breast cancer subtypes on the association analyses, subset analysis for estrogen receptor (ER+), progesterone receptor (PR+) and human epidermal growth factor receptor 2 (HER2+) breast cancer subtypes was carried out. Minimac dosages for Phase I-II and Phase III GWAS were converted to hard genotypes. Subset association analysis of ER+, PR+ and HER2+ breast cancer was calculated in PLINK using logistic regression adjusting for covariates PC1, PC2 and age. Permutation was performed for 1000 iterations to confirm the validity of the associations, with permutation *P*-value < 0.05 considered a valid association.

### Validation study

To select SNPs for validation study, logistic regression analysis was performed by including the effects of primary associated SNPs of a genomic locus in order to exclude SNPs that have the similar effects and to identify SNPs that are independently associated with breast cancer from the primary associated SNPs.

A total of 22 SNPs with *P*_meta < _1.0 × 10^−5^ that are not published previously to be associated with breast cancer were selected for validation study by using an independent samples group of 981 breast cases and 1,394 controls from the Aichi Cancer Centre, Japan. Genotyping of the SNPs were performed by using Multiplex Invader Assay. Considering the multiple testing for validation study, Bonferroni correction threshold at *P* < 5.00 × 10^−3^ was applied.

To evaluate the combined effects of discovery Phase I-II, Phase III GWAS and validation study, meta-analysis was performed using weighted inverse-variance^62^.

### Pathway and eQTL analysis

Meta-Analysis Gene-set Enrichment of variaNT Associations (MAGENTA, ver2.4)^67^ software was used to assess potential pathways that are associated with breast cancer by using GWAS and 1000 G imputed dataset (RSQ > 0.9). In brief, gene boundary between 110 kb upstream of the gene’s most extreme transcript start site and 40 kb downstream to the gene’s most extreme transcript end site was set. This boundary was suggested by a comprehensive study of putative functional regulatory element (cis-eQTLs) using expression data from human lymphoblastoid cell line. After assigning SNPs within the gene boundary, cumulative *P*-value of the SNPs within individual genes were calculated after correcting for confounders such as gene size, variant number and LD properties using step-wise multiple linear regression analysis. Gene-set enrichment analysis *P*-value was calculated by referring to a total of 3,217 gene-sets from Gene ontology, KEGG, PANTHER, Biocarta and Reactome databases. False discovery rate (*FDR* < 0.05) was used to evaluate the significance of associations of the pathway with breast cancer.

To assess expression quantitative trait loci (eQTL), correlation between SNP genotypes and expression of the nearby genes in a genomic locus was evaluated from the GTEx portal V8 (http://www.gtexportal.org/home/) focusing specifically on breast-mammary tissue. The eQTL analysis was expanded to include all 12 genome-wide level linked variants (LD > = 0.8 ASN 1 K genomes) due to potential differences in the database SNP repository.

### Weighted genetic risk score (wGRS)

wGRS analysis was carried out to evaluate the cumulative effects of genetic variants associated with breast cancer risk. A total of 12 SNPs that are with *P* < 5.0 × 10^−8^ from the whole-genome meta-analysis of Phase I + II and Phase III GWAS were utilized to establish the model. These SNPs include rs2540431 on chromosome 2q33.1, rs9862599 on 3q13.11, rs1838337 on 3q25.1, rs7701466 on 5p12, rs79160707 on 5q11.2, rs6900157 on 6q25.1, rs2912778 on 10q26.13, rs805583 on 12p11.22, rs10744856 on 12q24.21, rs3803662 and rs4784227 on 16q12.1, rs75286142 on 21q22.12. The estimate (weight) of each associated SNP was evaluated by multivariate logistic regression analysis after incorporating 12 SNPs into the model. The cumulative risk scores were calculated by multiplying the weight (estimate) of the SNPs with the number of risk alleles (0/1/2) of the SNPs carried by each of the individual, subsequently the sum of the scores were taken across the number of SNPs in the model. The risk scores were then classified into four different categories that derived from mean and standard deviation (SD); group 1, < mean-1SD; group 2, mean-1SD to mean; group 3 mean to mean + 1 SD; group 4, mean + 1 SD to mean + 2 SD and group 5 > mean + 2 SD. Odds ratio and 95% confidence interval was evaluated by using group 1 as reference. Validation of this model was carried out by genotyping the 12 SNPs using the sample groups from Aichi Cancer Centre. Similar categorization was performed to evaluate the validity of this model. Lastly, receiving operating characteristic (ROC) curve was plotted to observe how well this model could be used as prediction model for breast cancer.

## Supplementary information

Supp Table 1, Supp Table 2, Supp Table 3, Supp Table 4, Supp Table 5, Supp Table 6

Supplementary Information

## Data Availability

The summary statistics for the GWAS will be publicly available from the National Bioscience Database Center (NBDC) Human Database (https://humandbs.biosciencedbc.jp/en/). The genotype data of case subjects in BBJ_Phase I-II and case and control subjects in BBJ_Phase III are available at the Japanese Genotype-phenotype Archive (JGA; http://trace.ddbj.nig.ac.jp/jga/index_e.html) with accession codes JGAS00000000114 for the study and JGAD00000000123 for the genotype data. The other datasets generated during and/or analysed during the current study are available from the corresponding author on reasonable request.
