# Supplementary material for: Identification of two novel breast cancer loci through large-scale genome-wide association study in the Japanese population
Source: Sci Rep. 2019 Nov 22;9:17332. doi: 10.1038/s41598-019-53654-9 (PMC6874604; doi:10.1038/s41598-019-53654-9)
Supplement: Supplementary file 2 — Supplementary Information [file 41598_2019_53654_MOESM2_ESM.pdf]

Supplementary Information

Identification of two novel breast cancer loci through large-scale genome-wide association study in the Japanese population

Siew-Kee Low[1, 2, *], Yoon Ming Chin[1], Hidemi Ito[4, 6], Keitaro Matsuo[5, 6], Chizu Tanikawa[7], Koichi Matsuda[9], Hiroko Saito[10], Mika Sakurai-Yageta[11], Naoki Nakaya[11], Atsushi Shimizu[12], Satoshi S. Nishizuka[12], Taiki Yamaji[13], Norie Sawada[13], Motoki Iwasaki[13], Shoichiro Tsugane[14], Toshiro Takezaki[15], Sadao Suzuki[16], Mariko Naito[17, 18], Kenji Wakai[17], Yoichiro Kamatani[2], Yukihide Momozawa[3], Yoshinori Murakami[8], Johji Inazawa[19,21], Yusuke Nakamura[1], Michiaki Kubo[3], Toyomasa Katagiri[22], Yoshio Miki[10, 20]

[1]Cancer Precision Medicine Center, Japanese Foundation for Cancer Research, Tokyo, Japan
[2]Laboratory for Statistical Analysis, and [3]Laboratory for Genotyping Development, RIKEN Center for Integrative Medical Sciences, Yokohama, Japan
[4]Division of Cancer Information and Control, and [5]Division of Cancer Epidemiology and Prevention, Aichi Cancer Center Research Institute, Nagoya, Japan.
[6]Department of Epidemiology, Nagoya University Graduate School of Medicine
[7]Laboratory of Genome Technology, Human Genome Center, and [8]Division of Molecular Pathology, The Institute of Medical Science, The University of Tokyo, Tokyo, Japan
[9]Graduate school of Frontier Sciences, The University of Tokyo, Tokyo, Japan
[10]Department of Genetic Diagnosis, The Cancer Institute of JFCR, Tokyo, Japan
[11]Tohoku Medical Megabank Organization, Tohoku University, Sendai, Japan,
[12]Iwate Tohoku Medical Megabank Organization, Iwate Medical University, Iwate, Japan,
[13]Division of Epidemiology, [14]Center for Public Health Sciences, National Cancer Center, Tokyo, Japan
[15]Department of International Island and Community Medicine, Kagoshima University Graduate School of Medical and Dental Sciences, Kagoshima, Japan
[16]Department of Public Health, Nagoya City University Graduate School of Medical Sciences, Nagoya, Japan
[17]Department of Preventive Medicine, Nagoya University Graduate School of Medicine, Nagoya, Japan
[18]Department of Oral Epidemiology, Graduate School of Biomedical and Health Sciences, Hiroshima University, Hiroshima, Japan
[19]Department of Molecular Cytogenetics, and [20]Department of Molecular Genetics, Medical Research Institute, Tokyo Medical & Dental University, Tokyo, Japan
[21]Bioresource Research Center, Tokyo Medical & Dental University, Tokyo, Japan
[22]Division of Genome Medicine, Institute for Genome Research, Tokushima University, Tokushima, Japan

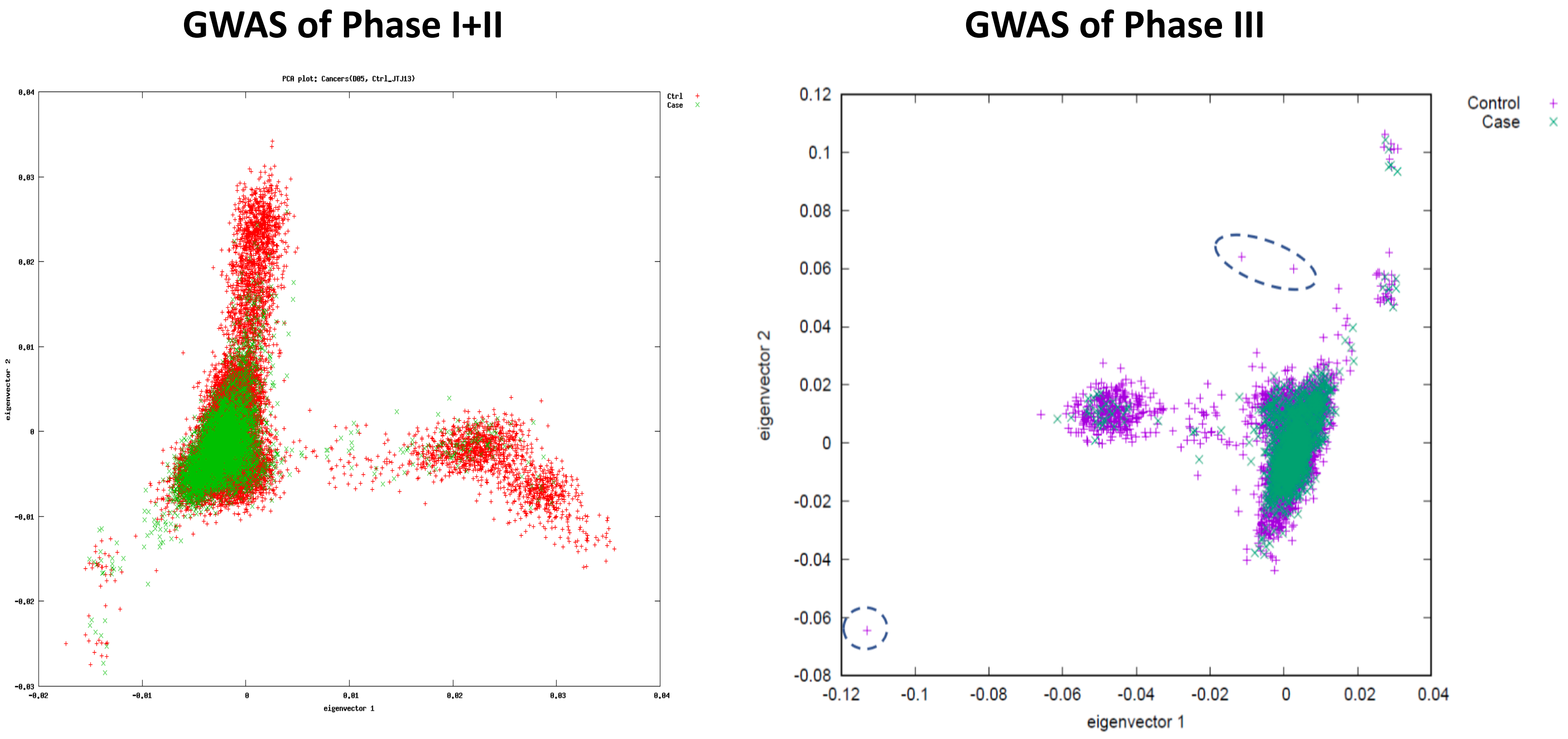

Supplementary Figure 1: Principal component analysis to evaluate population structure for GWAS of breast cancer in Japanese population. From the results of PCA analysis for GWAS Phase III, three control outliers were excluded from the study.

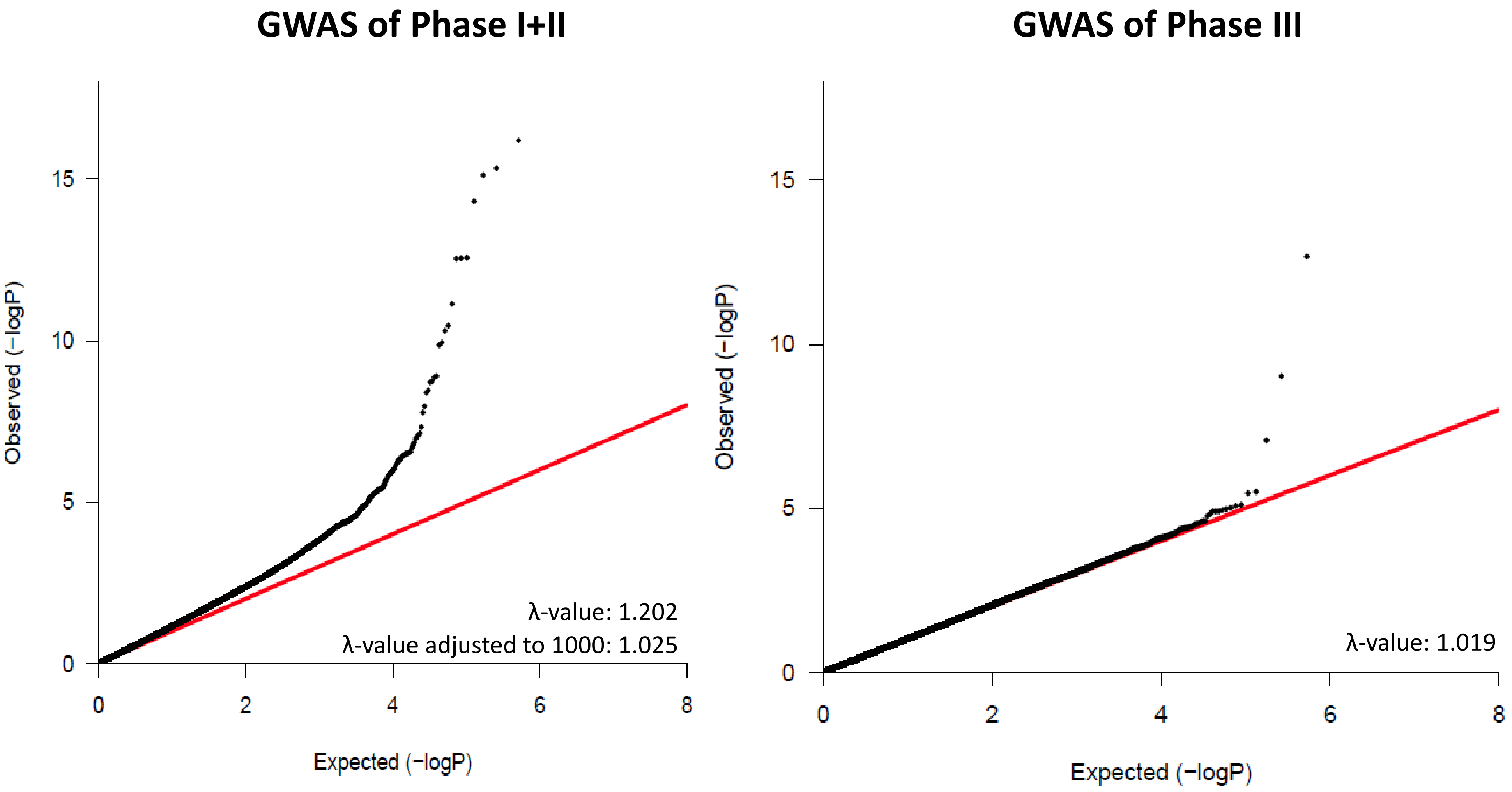

Supplementary Figure 2: Quantile-quantile plots for the GWAS of breast cancer.

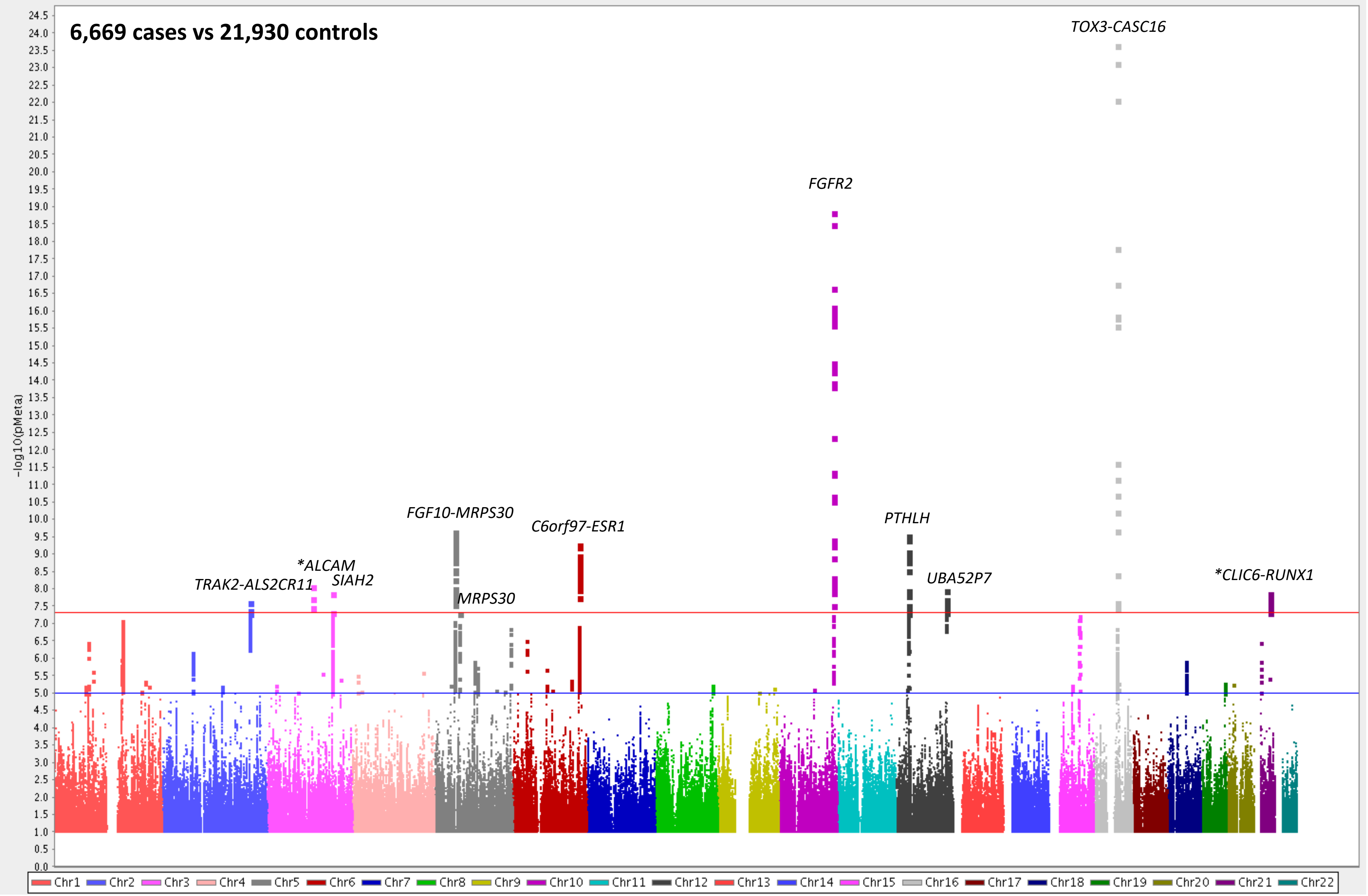

Supplementary Figure 3: Manhattan plot for genome-wide meta-analyses and 1000genome imputation analysis for the GWAS of breast cancer in Japanese population. *indicate novel loci identified from this study.

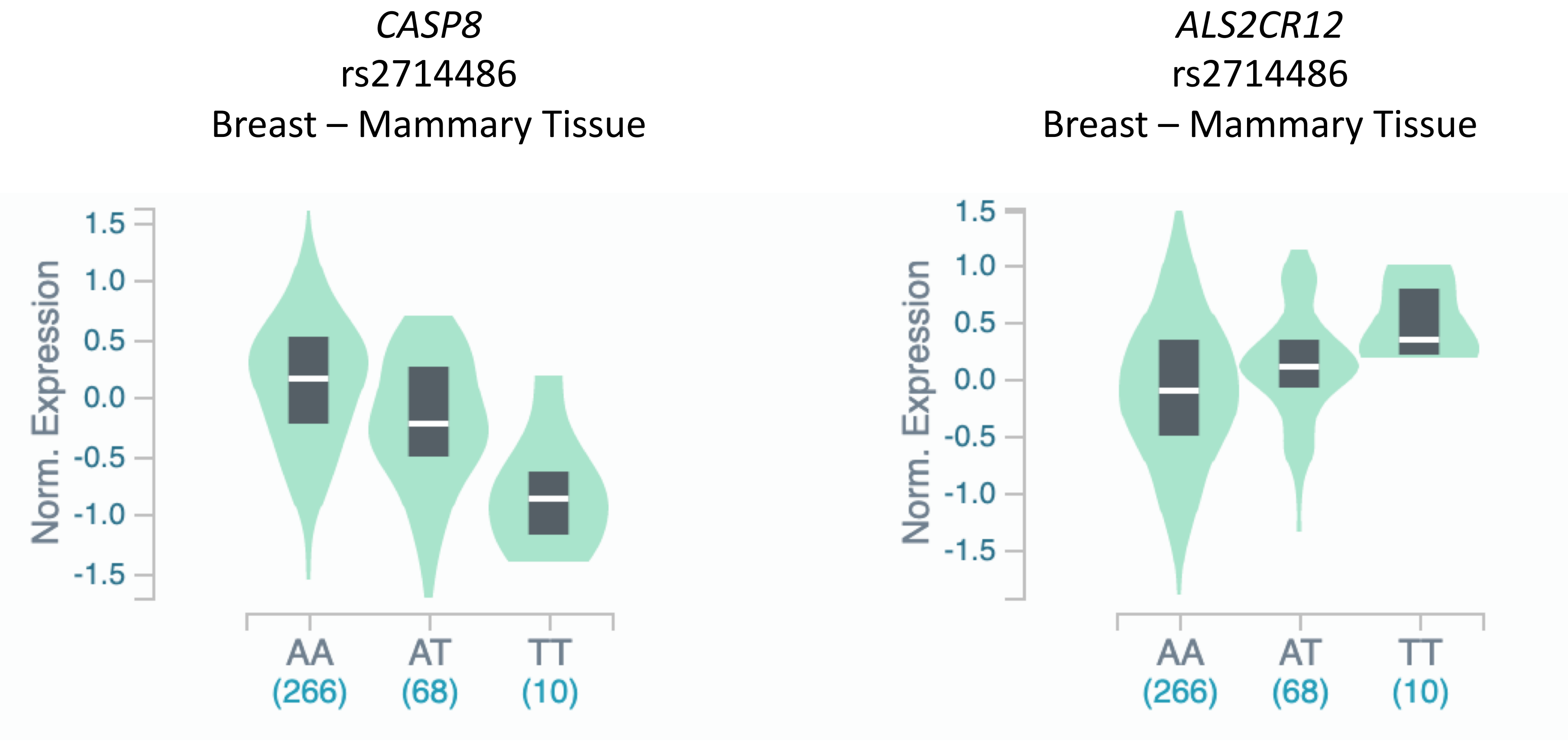

Gencode Id	Gene Symbol	Variant Id	SNP Id	P-Value	NES	Tissue
ENSG00000064012.21	*CASP8*	chr2_201384985_A_T_b38	rs2714486	4.20E-12	-0.44	Breast - Mammary Tissue
ENSG00000155749.12	*ALS2CR12*	chr2_201384985_A_T_b38	rs2714486	5.20E-07	0.35	Breast - Mammary Tissue

Supplementary Figure 4a: GTEx eQTL results of rs2540431-linked SNP rs2714486 in breast mammary tissue. rs2714486-A is the risk allele. Number of samples per genotype is stated in brackets.

CASP8
rs2540334
Breast – Mammary Tissue

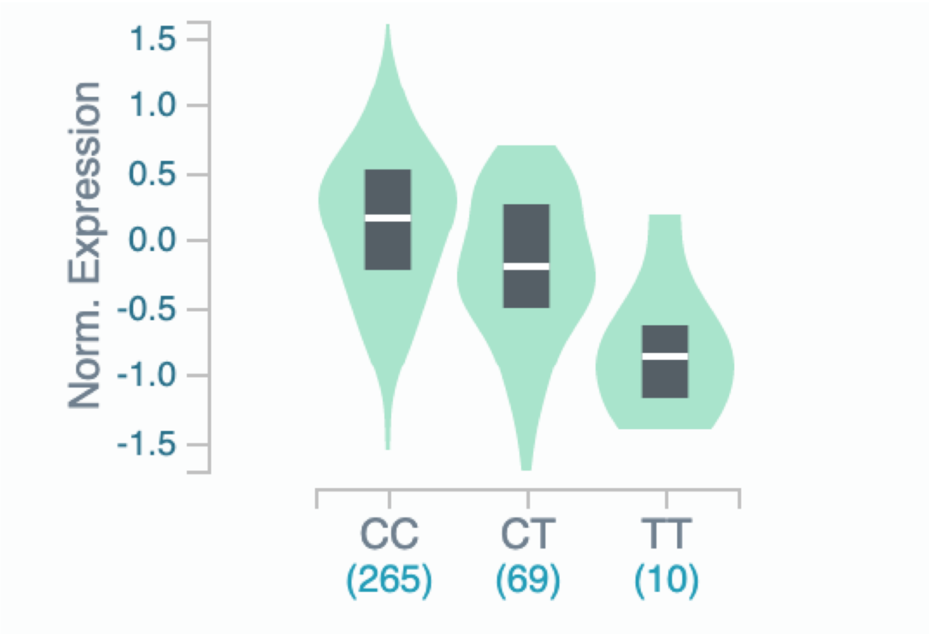

ALS2CR12
rs2540334
Breast – Mammary Tissue

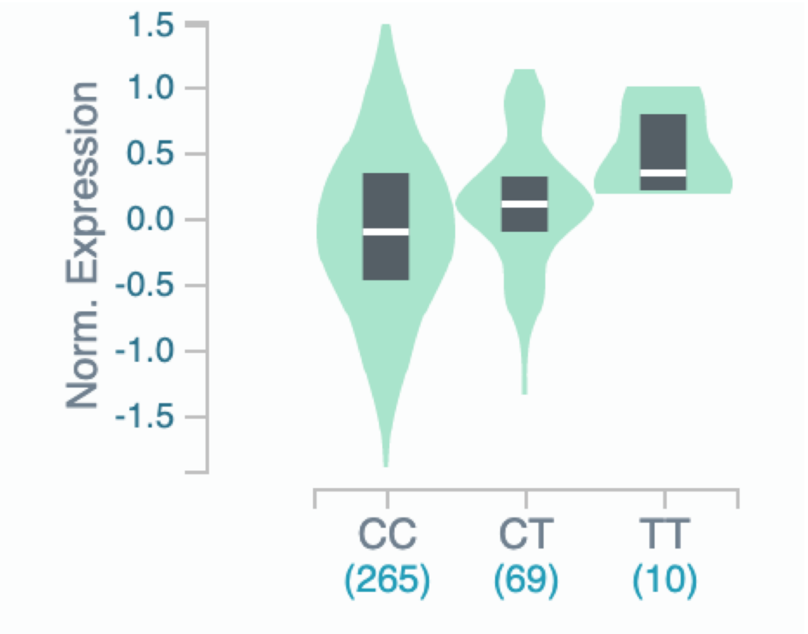

Gencode Id	Gene Symbol	Variant Id	SNP Id	P-Value	NES	Tissue
ENSG00000064012.21	CASP8	chr2_201406624_C_T_b38	rs2540334	6.90E-12	-0.43	Breast - Mammary Tissue
ENSG00000155749.12	ALS2CR12	chr2_201406624_C_T_b38	rs2540334	7.80E-07	0.34	Breast - Mammary Tissue

Supplementary Figure 4b: GTEx eQTL results of rs2540431-linked SNP rs2540334 in breast mammary tissue. rs2540334-T is the risk allele. Number of samples per genotype is stated in brackets.

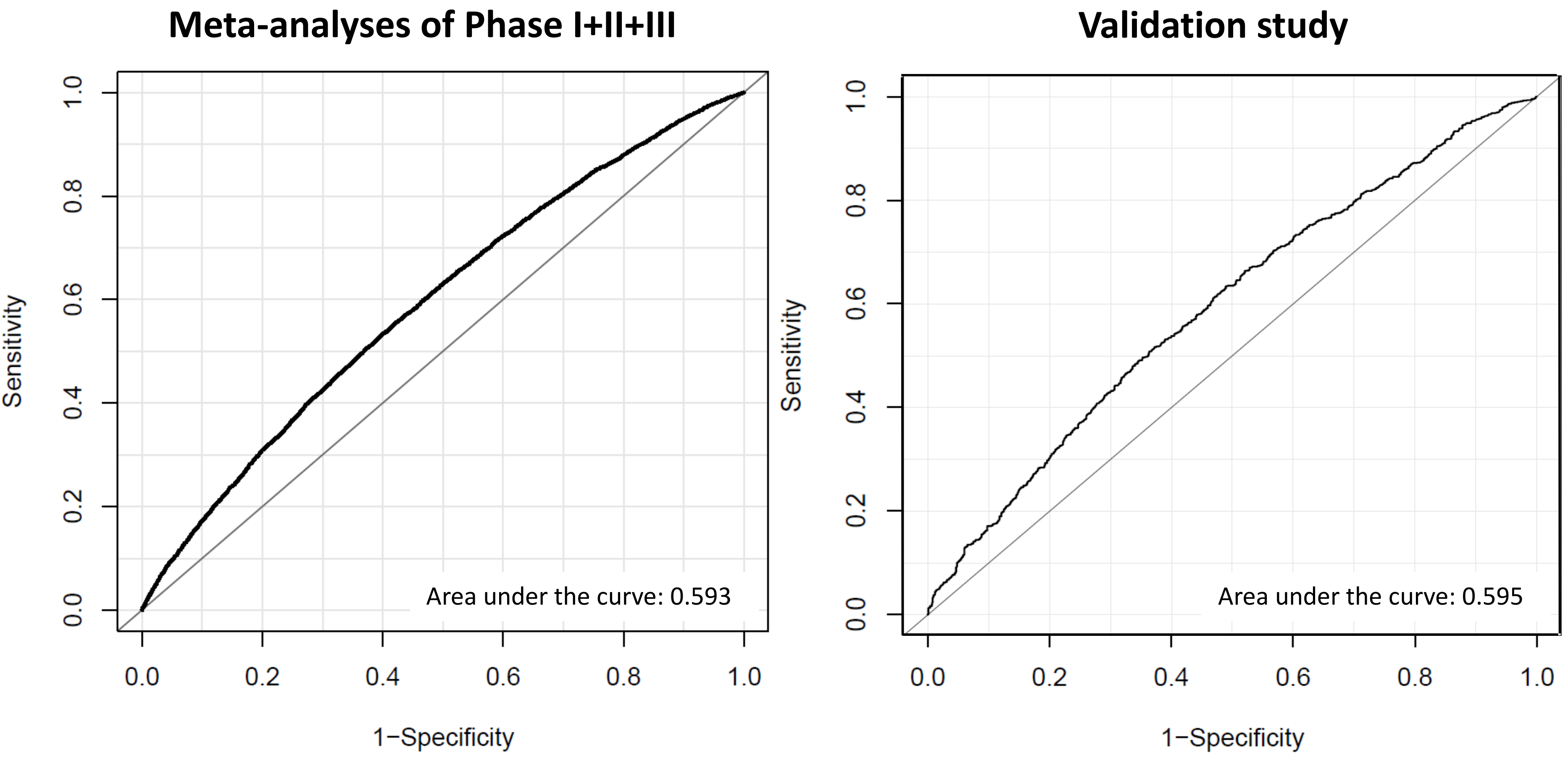

Supplementary Figure 5: ROC curve of wGRS prediction model to evaluate the cumulative effects of 12 significantly associated SNPs.
